# Assessment of Radioactive Materials and Heavy Metals in the Surface Soil around the Bayanwula Prospective Uranium Mining Area in China

**DOI:** 10.3390/ijerph14030300

**Published:** 2017-03-14

**Authors:** Haribala Bai, Bitao Hu, Chengguo Wang, Shanhu Bao, Gerilemandahu Sai, Xiao Xu, Shuai Zhang, Yuhong Li

**Affiliations:** 1 School of Nuclear Science and Technology, Lanzhou University, Lanzhou 730000, China; harbl12@lzu.edu.cn (H.B.); hubt@lzu.edu.cn (B.H.); 2 The Inner Mongolia Autonomous Region Comprehensive Center for Disease Control and Prevention, Huhhot 010031, China; wangcgcdc@163.com (C.W.); kaki2006119020@163.com (G.S.); foxxiao1982@163.com (X.X.); zsacml@126.com (S.Z.); 3 College of Geographical Science, Inner Mongolia Normal University, Huhhot 010022, China; baoshanhu@imnu.edu.cn

**Keywords:** soil, radionuclide, heavy metal, uranium mining area, health risk

## Abstract

The present work is the first systematic and large scale study on radioactive materials and heavy metals in surface soil around the Bayanwula prospective uranium mining area in China. In this work, both natural and anthropogenic radionuclides and heavy metals in 48 surface soil samples were analyzed using High Purity Germanium (HPGe) γ spectrometry and inductively coupled plasma-mass spectrometry (ICP-MS). The obtained mean activity concentrations of ^238^U, ^226^Ra, ^232^Th, ^40^K, and ^137^Cs were 25.81 ± 9.58, 24.85 ± 2.77, 29.40 ± 3.14, 923.0 ± 47.2, and 5.64 ± 4.56 Bq/kg, respectively. The estimated average absorbed dose rate and annual effective dose rate were 76.7 ± 3.1 nGy/h and 83.1 ± 3.8 **μ**Sv, respectively. The radium equivalent activity, external hazard index, and internal hazard index were also calculated, and their mean values were within the acceptable limits. The estimated lifetime cancer risk was 3.2 × 10^−4^/Sv. The heavy metal contents of Cr, Ni, Cu, Zn, As, Cd, and Pb from the surface soil samples were measured and their health risks were then assessed. The concentrations of all heavy metals were much lower than the average backgrounds in China except for lead which was about three times higher than that of China’s mean. The non-cancer and cancer risks from the heavy metals were estimated, which are all within the acceptable ranges. In addition, the correlations between the radionuclides and the heavy metals in surface soil samples were determined by the Pearson linear coefficient. Strong positive correlations between radionuclides and the heavy metals at the 0.01 significance level were found. In conclusion, the contents of radionuclides and heavy metals in surface soil around the Bayanwula prospective uranium mining area are at a normal level.

## 1. Introduction

Radiation exposure and heavy metal pollution around uranium mining areas have captured worldwide public attention for several decades [1,2,3,4]. The intensive uranium exploitation and the inappropriate management of the residues have had a harmful impact on the environment [4,5,6]. In recent decades, the dose contribution from technologically enhanced naturally occurring radioactive materials is increasing [7,8]. The worldwide annual effective dose to the public from natural radiation exposure is 2.4 mSv [9], while it is 3.1 mSv in China, which increased from 2.3 mSv in 1990s [10,11,12]. The demand for uranium resources in China is increasing with the development of nuclear power industries [9,13,14] and the rising price of uranium internationally [15]. Consequently, the activities on exploiting uranium ores and their hydrometallurgy processes were heavily strengthened and there are also some reports concerning the environmental contamination around uranium mines [16]. However, there have been few specific studies related to radionuclides and heavy metals assessment from uranium mining areas in China, especially around prospective uranium mining areas. A pre-mining study on radiation levels and heavy metals around uranium mining areas could establish a baseline database on the environmental radiation levels and become an essential reference guide for the future [17]. The aim of this study was to establish the radioactive materials and heavy metals contents from the surface soil around the Bayanwula uranium pre-mining area in China due to the lack of published environmental data, to assess the radiation and heavy metals risk for local residents, and to investigate the correlations between the radionuclides and heavy metals.

## 2. Materials and Methods

### 2.1. Study Area

**Figure 1:**
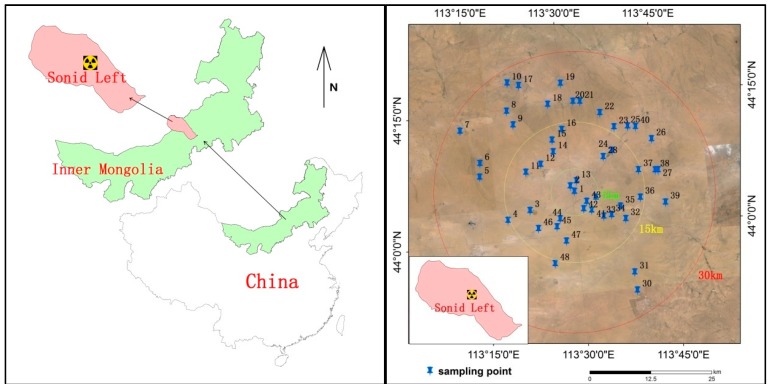
The map of the Bayanwula uranium mining area and sampling locations.

The Bayanwula uranium mining region is located in the central part of the Sonid Left district, which is in the northwest part of the XilinGol prairie of Inner Mongolia in China. The study area is about 30 km north of the capital of Sonid Left. The altitude ranges from 1040 m to 1255 m. There are around 34,000 residents in Sonid Left. This region has a continental climate with a warm summer and cold winter. The average annual precipitation is less than 200 mm. The study area is characterized by grassland, not cultivated, and no industries. The sampling was carried out in June 2015 prior to the uranium mining activities. The map of the mining area and sampling locations are shown in Figure 1, in which the sampling locations were mapped using the software ESRI Arc GIS desktop 10.1 (Environmental Systems Research Institute, Inc., Redlands, CA, USA) based on the coordinates determined by the Global Positioning System (GPS).

### 2.2. Sampling and Preparation

As shown in Figure 1, a total of 48 surface soil samples were collected within about a 30 km radius from the center of the mining area. At each sampling location, a square area of 1 m^2^ was marked out. Then four samples were collected from the surface layer (up to 10 cm) of the four corners of the square area (1 m × 1 m) using a stainless steel cylindrical sampler (Ø10 cm × H10 cm), mixed, and placed in a labeled polythene bag after removing impurities such as stones, gravels, and roots. In the laboratory, each sample was dried in an oven at 100 °C for more than 24 h to remove the moisture content, homogenized, and was separated into two parts. One of them was sieved through a 0.25 mm mesh. A sample of 338.0 g was weighed and sealed in an airtight polythene (Ø75 cm × H70 cm) cylindrical container and left for more than 30 days to allow ^226^Ra and its decay products to reach secular equilibrium before further gamma-ray measurement. The concentrations of ^238^U, ^226^Ra, ^232^Th, ^40^K, and ^137^Cs were determined by a HPGe γ-ray spectrometry system (Oak Ridge Technology & Engineering Cooperation, Oak Ridge, TN, USA).

The other part was sieved through 0.150 mm mesh and weighed 0.2–0.5 g with accuracy up to 0.1 mg. They were then digested with a concentrated acid mixture (HNO_3_, HF, and HClO_4_) (Analytical reagent, EMD Millipore Corporation, Darmstadt, Germany). The solution was transferred to a 25 milliliter volumetric flask. The content of 7 elements (Cr, Ni, Cu, Zn, As (non-metal trace element), Cd, and Pb) was determined by inductively coupled plasma-mass spectrometry (ICP-MS) (Agilent Technologies Inc., Santa Clara, CA, USA). Lower limits of detection (LLDs) were determined as 10 μg/kg for Cr, 13 μg/kg for Ni, 13 μg/kg for Cu, 8 μg/kg for Zn, 3 μg/kg for As, 0.3 μg/kg for Cd, and 7 μg/kg for Pb in dry soil weight.

HPGe γ-ray spectrometry system employed to carry out the radioactivity measurements was based on a high-purity germanium p-type coaxial photon detector made by Oak Ridge Technology & Engineering Cooperation (ORTEC). The detector relative efficiency exceeded 32% while the resolution was better than 1.82 keV at 1.33 MeV ^60^Co. The γ spectrum of 40 keV–3 MeV was acquired and analyzed using the software Gamma vision (6.01) (Oak Ridge Technology & Engineering Cooperation, Oak Ridge, TN, USA) and a 8192 multichannel analyzer (Oak Ridge Technology & Engineering Cooperation, Oak Ridge, TN, USA). The whole detector system was placed inside a 10 cm lead layer shield. Before and after all sample counting, the backgrounds were measured and were subtracted from the corresponding photopeaks. The energy and efficiency calibrations of the counting system were performed using γ sources of ^238^U, ^234^Th, ^226^Ra, ^40^K, and ^137^Cs with the same size of each sample. It took 86,400 s to reduce the counting statistical error for each measurement. The activity concentrations of ^238^U, ^232^Th, ^226^Ra, ^40^K, ^137^Cs in the soil samples were determined in Bq/kg dry weight. The activity concentration of ^238^U was derived from ^234^Th (63.3 keV). The ^232^Th in the soil samples was derived from ^212^Pb (238.6 keV), ^208^Tl (538.2 keV), and ^228^Ac (911.2 keV). The ^226^Ra activity was determined by its daughter radionuclides ^214^Pb at 351.9 keV and ^214^Bi at 609.3 keV. The activity concentrations of ^40^K and ^137^Cs were derived from the photopeaks of 1460.8 and 661.7 keV, respectively. The minimum detectable activity for each radionuclide was determined from the HPGe γ-ray spectrometry system and samples for the counting time of 86,400 s, and was estimated to be 3.7 Bq/kg for ^238^U, 0.1 Bq/kg for ^232^Th, 0.1 Bq/kg for ^226^Ra, 1.7 Bq/kg for ^40^K, and 0.01 Bq/kg for ^137^Cs.

### 2.3. Radiation Hazard Index Calculation

The natural radioactivity of building materials is mainly from the ^238^U series, ^232^Th series, and ^40^K. As 98.5% of the radiological effects of the uranium series are produced by radium and its daughter products, the contribution from ^238^U has been replaced with the decay product ^226^Ra [18,19]. Therefore, the radiation hazard indices are usually determined by the activity concentrations of ^226^Ra, ^232^Th, and ^40^K.

#### 2.3.1. Absorbed γ Dose Rate in Air

The absorbed γ dose rate (nGy/h) in air at 1 m above the ground for radionuclides (^238^U series, ^232^Th series, and ^40^K) uniformly distributed on the ground was computed by following Equation (1) [9].

(1) $$D=0.462\times A_{\mathrm{Ra}}+0.604\times A_{\mathrm{Th}}+0.0417\times A_{K}$$

where A_Ra_, A_Th_, and A_K_ are the activity concentrations of ^226^Ra, ^232^Th, and ^40^K (Bq/kg), respectively.

#### 2.3.2. Annual Effective Dose

The annual effective dose is presented to express the irradiated dose of the human body from natural existing radionuclides in the earth’s crust soil. It is expressed [9] by following Equation (2).

(2) $$\mathrm{AED}=D\times 8760\times 0.2\;\left(\mathrm{or}\;0.8\right)\times 0.7\times 10^{-3}$$

where AED is annual effective dose (μSv/y); D is γ dose rate (nGy/h); the coefficient 0.7 Sv/Gy is for the conversion coefficient from the absorbed dose in air to the effective dose received by adults; 0.2 for the outdoor occupancy factor; 8760 hour/year is equal to 365 days × 24 h per year.

#### 2.3.3. Radium Equivalent Activity and External Hazard Index

Both the radium equivalent activity (Ra_eq_) and the external hazard index (H_ex_) were equally used to evaluate the effect of the external γ radiation on human beings. The radium equivalent activity and external hazard index were calculated by Equations (3) and (4). The Ra_eq_ should not exceed 370 Bq/kg and the H_ex_ should be less than unity [10].

(3) $${\mathrm{Ra}}_{\mathrm{eq}}=A_{\mathrm{Ra}}+1.43\times A_{\mathrm{Th}}+0.077\times A_{K}$$

(4) $$H_{\mathrm{ex}}=A_{\mathrm{Ra}}/370+A_{\mathrm{Th}}/259+A_{K}/4810$$

#### 2.3.4. Internal Hazard Index

The internal hazard index (H_in_) was introduced to describe the hazard of radon and its short-lived products in building materials, given by Equation (5) and recommended to be less than unity [10].

(5) $$H_{\mathrm{in}}=A_{\mathrm{Ra}}/185+A_{\mathrm{Th}}/259+A_{K}/4810$$

#### 2.3.5. Lifetime Cancer Risk

The lifetime cancer risk (LTCR) was obtained by Equation (6) [11,12]:

(6) LTCR=AED×DL×RFSE

where DL is the duration of lifetime, 70 years; and RFSE is the risk factor for stochastic effects of the common population, 0.055/Sv [12].

### 2.4. Health Risk Assessment of Heavy Metals

Human beings are exposed to soil metals through the ingestion and inhalation of dust particles through the mouth and nose, and dermal contact [20,21]. The health risk assessment model used in this study was developed by the US Environmental Protection Agency [22,23]. The doses are calculated as follows:

(7) $$D_{\mathrm{ing}}=\frac{C\times \mathrm{IngR}\times \mathrm{EF}\times \mathrm{ED}}{\mathrm{BW}\times \mathrm{AT}}\times 10^{-6}$$

(8) $$D_{\mathrm{inh}}=\frac{C\times \mathrm{InhR}\times \mathrm{EF}\times \mathrm{ED}}{\mathrm{PEF}\times \mathrm{BW}\times \mathrm{AT}}$$

(9) $$D_{\mathrm{dermal}}=\frac{C\times \mathrm{SA}\times \mathrm{SL}\times \mathrm{ABS}\times \mathrm{EF}\times \mathrm{ED}}{\mathrm{BW}\times \mathrm{AT}}\times 10^{-6}$$

where D_ing_, D_inh_, D_dermal_ are the average daily intake through ingestion, inhalation, and dermal absorption in mg/(kg·day), C is the concentration of metal in the soil (mg/kg), IngR and InhR are the ingestion and inhalation rate of soil, respectively (mg/day, m^3^/day), EF is the exposure frequency (day/year), and ED is the exposure duration (year). SA is the exposed skin area (cm^2^), SL is the skin adherence factor, ABS is the dimensionless dermal absorption factor, PEF is the particle emission factor in m^3^/kg, BW is the average body weight (kg), and AT is the average time (day). The doses calculated for each element and exposure pathway are subsequently divided by the corresponding reference dose (RfD) (mg/(kg·day)) to yield a hazard quotient (HQ) (or non-cancer risk), whereas for carcinogens, the dose is multiplied by the corresponding slope factor (SF) (mg/(kg·day))^−1^ to produce a level of cancer risk. The hazard index (HI) is then the sum of HQ [24]. If HI < 1, it is believed that there is no significant risk of non-carcinogenic effects and the magnitude of risk increases as HI increases [23]. Carcinogenic risk is used to estimate the probability of an individual developing any type of cancer from the lifetime exposure to carcinogenic hazards. The acceptable risk for regulatory purposes is in the range of 10^−6^–10^−4^ [20]. These values indicate that one additional case in a population of 1 million to one in 10,000 people is acceptable. In this study, hazard index methods and cancer risk methods were used to assess health risks of metal exposure to children and adults in the Bayanwula uranium pre-mining area. The detailed corresponding parameters are presented in Table 1 [20,21,23,25].

## 3. Results and Discussion

### 3.1. Radionuclides

The activity concentrations of radionuclides (^238^U, ^232^Th, ^226^Ra, ^40^K, and ^137^Cs) in 48 surface soil samples around the Bayanwula prospective uranium mining area are presented in Table 2. The mean values of ^238^U, ^232^Th, and ^226^Ra are lower than the China and world mean values. However, the mean value of ^40^K was around two times higher than both the worldwide and China’s average of 412 [6] and 580.0 Bq/kg [26], respectively. The activity concentration of ^137^Cs was 5.64 Bq/kg, which was the anthropogenic radionuclide from nuclear weapon tests or nuclear power accidents. The absorbed γ dose rate in air, annual effective dose, hazard indices, and lifetime cancer risk calculated from radionuclides in soil samples are shown in Table 3. The calculated mean outdoor γ dose rates was 76.7 nGy·h^−1^, which was higher than the world average of 60 nGy/h [6] and the Chinese mean value of 62.8 nGy/h [26]. The mean value of radium equivalent activity was 138.0 Bq/kg, lower than the reference value of 370 Bq/kg. The external and internal hazard indices did not exceed unity, which indicates that the γ radiation from the soil was at a safe level. The lifetime cancer risk was 3.2 × 10^−4^/Sv, which was also at a very low level.

### 3.2. Heavy Metals

The contents of heavy metals (Cr, Ni, Cu, Zn, As, Cd, and Pb) in surface soil from the prospective uranium mining area, background values of Inner Mongolia, mean values of China [27,28], and China soil guidelines [29] are also given in Table 2. The mean concentrations of Cr, Ni, Cu, Zn, As, and Cd are much lower than both the national mean backgrounds and the grade І soil quality standard (This level is mainly applicable to the national nature reserve except for the high background areas). However, the concentration of Pb is much higher than China’s background value and within the grade II soil quality standard (The level is mainly applied to general farmland, vegetable land, tea garden, orchard, pasture, and other soil; the soil quality basically could not cause harm and pollution to plants and the environment). The results of the health risk assessment of the heavy metals in the soil around the study area are listed in Table 4 for children and Table 5 for adults. For non-cancer risk, the ingestion dose of the heavy metals is significant for children and adults. The non-cancer risk of the heavy metals for children is higher than that for adults. The hazard indices (HIs) decrease in the order of Pb > Cr > As > Ni > Cu > Cd > Zn for both children and adults are all lower than unity. For cancer risk, Cr, Ni, As and Cd were considered in this study. The cancer risk from the heavy metals is much lower than the acceptable range of 10^−4^. It can be clearly seen from the tables that the non-cancer risk is more important than the cancer risk for both children and adults. These results indicate that both the cancer and non-cancer risks for the children and adults living around the Bayanwula prospective uranium mining region are all at acceptable levels.

### 3.3. Correlation Analysis

The correlations between the natural radionuclides and the heavy metals in the surface soil samples were performed using the SPSS computer package, Version 19 for Windows. The statistical significance of the Pearson correlation was determined by the *t* test [30,31]. If a value was close to zero, there was no association between the two elements. The terms “weak”, “moderate”, and “strong” were presented for correlation coefficients of 0.2–0.4, 0.4–0.6, and >0.6, respectively [31]. The alpha level for testing significance was set at 0.01 and 0.05. The Pearson correlations of the heavy metals and radionuclides are shown in Table 6. It was found that ^238^U was weakly positively correlated with ^232^Th and ^226^Ra at the 0.05 significance level. A strong positive correlation between ^232^Th and ^226^Ra at the 0.01 significance level was present. Both the radionuclides ^232^Th and ^226^Ra moderately positively correlated with Cr and Zn, and weakly correlated with ^40^K and Ni. There were also strong positive correlations between heavy metals: Cr and Zn, Ni and Cu, and Cu and Zn. These strong correlations among metals and radionuclides suggest their common origin. However, there are observed moderate or strong negative correlations between the radionuclide ^40^K with Ni, Cu, and Zn at the 0.01 significance level. Additionally, it was found that no correlations exist between the radionuclides and heavy metals, i.e., Cr and ^40^K. The absence of correlations could be explained by the mutual independence or different behavior of the elements.

## 4. Conclusions

The radionuclides (^238^U, ^232^Th, ^226^Ra, ^40^K, ^137^Cs) and heavy metals were measured in 48 surface soil samples from the Bayanwula prospective uranium mining area in China. Activity concentrations of ^238^U, ^232^Th, and ^226^Ra were lower than the world average except for ^40^K. The values obtained were within the acceptable limits. The annual effective dose and various radiation hazard indices indicate that there is low radiological risk to the local populations around the uranium mining area. The contents of the heavy metals Cr, Ni, Cu, Zn, As, and Cd were within the Chinese soil guidelines Grade І except for Pb, which was about three times higher than the average of China. The non-cancer risk index and cancer risk were estimated to be less than the acceptable limits. The risks of heavy metals for children are all higher than that for adults. A strong positive correlation between radionuclides and heavy metals at the 0.01 significance level was found which suggests their common origin. The correlation study also indicated negative and weak correlations between the radionuclides and heavy metals. This study established the baseline information regarding the natural, artificial radioactivity, and heavy metals status around the Bayanwula prospective uranium mining area in China. To the best of our knowledge, this is the first systematic and large scale study on radiation levels around prospective uranium mining areas in China. These background data could be an important reference for public environmental concerns.

## Figures and Tables

**Table 1:** Parameters used to evaluate the exposure risk of soil metals.

Parameter	Symbol	Unit	Value
Soil ingestion rate	IngR	mg/day	200 (child), 100 (adult)
Exposure frequency	EF	day/year	350
Soil inhalation rate	InhR	m^3^/day	7.6 (child), 20 (adult)
Exposure duration	ED	year	70 [6 (child) for non-cancer effects]
Skin area	SA	cm^2^	860 (child), 1530 (adult)
Skin adherence factor	SL	mg·cm^2^	0.2 (child), 0.07 (adult)
Dermal absorption factor	ABS	unitless	0.006 (Pb), 0.14 (Cd), 0.1 (Cu), 0.02 (Zn), 0.05 (Hg), 0.03 (As)
Particle emission factor	PEF	m^3^/kg	1.36 × 10^9^
Body weight	BW	kg	15 (child), 70 (adult)
Averaging time	AT	day	ED × 365 days for non-carcinogens
			70 × 365 days for carcinogens
Chronic reference dose	RfD	mg·kg^−1^·day^−1^	Ingestion RfD: 3.00 × 10^−3^ (Cr), 2.00 × 10^−2^ (Ni), 4.00 × 10^−2^ (Cu), 3.00 × 10^−1^ (Zn), 3.00 × 10^−4^ (As), 1.00 × 10^−3^ (Cd), 3.50 × 10^−3^ (Pb)
Carcinogenic slope factor	SF	(mg·kg^−1^·day^−1^)^−1^	Inhalation RfD: 2.86 × 10^−5^ (Cr), 2.06 × 10^−2^ (Ni), 4.02 × 10^−2^ (Cu), 3.00 × 10^−1^ (Zn), 3.01 × 10^−4^ (As), 3.25 × 10^−3^ (Pb)
			Dermal RfD: 6.00 × 10^−5^ (Cr), 5.40 × 10^−5^ (Ni), 1.20 × 10^−2^ (Cu), 6.00 × 10^−6^ (Zn), 1.23 × 10^−4^ (As), 1.00 × 10^−5^ (Cd), 5.25 × 10^−4^ (Pb)
			Ingestion SF: 1.5 (As)
			Inhalation SF: 4.20 × 10^1^ (Cr), 8.40 × 10^−1^ (Ni), 1.51 × 10^1^ (As), 6.30 (Cd)
			Dermal SF: 3.66 (As)

**Table 2:** The contents of radionuclides (Bq/kg) and heavy metals (mg/kg) in surface soil samples around the Bayanwula prospective uranium mining area.

No.	^238^U	^232^Th	^226^Ra	^40^K	^137^Cs	Cr	Ni	Cu	Zn	As	Cd	Pb
1	20 ± 2 ^a^	30 ± 3	26 ± 3	887 ± 80	9.6 ± 1.0	12.6 ± 0.1^b^	5.4 ± 0.1	4.8 ± 0.0	16.7 ± 0.2	0.9 ± 0.0	0.010 ± 0.004	76.5 ± 1.1
2	16 ± 2	28 ± 3	24 ± 2	885 ± 80	2.2 ± 0.2	13.4 ± 0.2	5.3 ± 0.0	4.7 ± 0.0	15.0 ± 0.4	0.9 ± 0.0	0.006 ± 0.002	73.4 ± 0.9
3	25 ± 2	28 ± 3	24 ± 2	942 ± 85	4.9 ± 0.5	12.3 ± 0.2	5.3 ± 0.0	4.9 ± 0.0	15.9 ± 0.1	1.1 ± 0.0	0.006 ± 0.001	72.5 ± 0.5
4	21 ± 2	28 ± 3	24 ± 2	927 ± 83	3.0 ± 0.3	11.0 ± 0.2	4.8 ± 0.0	4.4 ± 0.1	15.0 ± 0.1	1.1 ± 0.0	0.002 ± 0.002	41.6 ± 0.7
5	44 ± 4	27 ± 3	23 ± 2	969 ± 87	13.9 ± 1.4	12.8 ± 0.0	5.6 ± 0.0	5.0 ± 0.1	17.2 ± 0.2	1.2 ± 0.0	0.006 ± 0.002	67.2 ± 0.3
6	23 ± 2	30 ± 3	26 ± 2	933 ± 84	5.1 ± 0.5	14.3 ± 0.5	5.8 ± 0.1	5.5 ± 0.1	17.3 ± 0.3	1.2 ± 0.0	0.004 ± 0.001	68.3 ± 1.1
7	22 ± 2	28 ± 3	25 ± 2	969 ± 87	6.2 ± 0.6	17.4 ± 0.1	5.1 ± 0.0	4.8 ± 0.0	19.2 ± 0.1	1.0 ± 0.0	0.005 ± 0.003	93.8 ± 0.6
8	34 ± 3	32 ± 3	26 ± 2	964 ± 87	2.2 ± 0.2	16.6 ± 0.2	6.0 ± 0.0	5.8 ± 0.0	20.0 ± 0.2	1.1 ± 0.0	0.005 ± 0.001	96.0 ± 0.3
9	23 ± 2	30 ± 3	26 ± 3	984 ± 89	8.4 ± 0.8	16.4 ± 0.2	4.8 ± 0.1	4.4 ± 0.1	17.4 ± 0.4	0.9 ± 0.0	0.003 ± 0.001	73.5 ± 3.7
10	22 ± 2	35 ± 3	29 ± 3	929 ± 84	6.2 ± 0.6	17.3 ± 0.2	5.3 ± 0.1	4.9 ± 0.1	18.2 ± 0.3	1.2 ± 0.0	0.007 ± 0.004	88.2 ± 1.5
11	29 ± 3	20 ± 2	17 ± 2	977 ± 88	10.0 ± 1.0	15.4 ± 0.2	4.6 ± 0.0	4.3 ± 0.0	16.3 ± 0.1	1.1 ± 0.0	0.016 ± 0.001	73.0 ± 0.2
12	14 ± 1	28 ± 3	22 ± 2	875 ± 79	8.8 ± 0.9	14.4 ± 0.1	6.0 ± 0.1	5.5 ± 0.1	16.3 ± 0.2	1.0 ± 0.0	0.012 ± 0.006	69.7 ± 1.9
13	25 ± 2	31 ± 3	29 ± 3	852 ± 77	6.3 ± 0.6	13.7 ± 0.1	5.9 ± 0.0	5.5 ± 0.0	16.6 ± 0.1	1.0 ± 0.0	0.010 ± 0.003	67.1 ± 0.2
14	18 ± 2	29 ± 3	24 ± 2	937 ± 84	15.5 ± 1.6	16.9 ± 0.5	4.6 ± 0.1	4.6 ± 0.2	18.8 ± 0.8	0.9 ± 0.0	0.009 ± 0.003	70.3 ± 5.2
15	13 ± 1	29 ± 3	26 ± 2	984 ± 89	4.9 ± 0.5	17.5 ± 0.1	5.1 ± 0.1	4.6 ± 0.0	17.5 ± 0.2	1.1 ± 0.0	0.004 ± 0.003	70.1 ± 1.7
16	24 ± 2	32 ± 3	26 ± 3	967 ± 87	1.4 ± 0.1	17.6 ± 0.1	6.0 ± 0.1	5.4 ± 0.1	18.3 ± 0.3	1.2 ± 0.0	0.001 ± 0.001	71.1 ± 0.6
17	37 ± 4	36 ± 3	32 ± 3	866 ± 78	1.5 ± 0.2	15.4 ± 0.4	8.7 ± 0.1	7.9 ± 0.1	23.2 ± 0.3	1.1 ± 0.0	0.009 ± 0.004	81.1 ± 0.5
18	58 ± 6	33 ± 3	27 ± 3	919 ± 83	3.2 ± 0.3	12.9 ± 0.2	6.3 ± 0.0	6.2 ± 0.1	19.9 ± 0.5	1.3 ± 0.0	0.009 ± 0.001	72.5 ± 0.3
19	17 ± 2	30 ± 3	23 ± 2	807 ± 73	4.2 ± 0.4	12.2 ± 0.4	6.8 ± 0.1	6.8 ± 0.1	19.8 ± 0.3	1.2 ± 0.0	0.020 ± 0.004	70.6 ± 1.7
20	30 ± 3	28 ± 3	24 ± 2	934 ± 84	15.6 ± 1.6	15.0 ± 0.1	5.8 ± 0.1	5.5 ± 0.1	19.8 ± 0.0	1.2 ± 0.0	0.011 ± 0.001	78.0 ± 0.7
21	24 ± 2	32 ± 3	27 ± 3	929 ± 84	0.6 ± 0.1	16.2 ± 0.3	5.9 ± 0.1	5.9 ± 0.1	19.3 ± 0.5	1.3 ± 0.1	0.004 ± 0.003	65.0 ± 0.6
22	27 ± 3	31 ± 3	26 ± 3	957 ± 86	4.1 ± 0.4	14.6 ± 0.1	5.7 ± 0.1	5.4 ± 0.1	18.4 ± 0.3	1.2 ± 0.0	0.006 ± 0.004	75.1 ± 0.4
23	31 ± 3	30 ± 3	27 ± 3	955 ± 86	14.6 ± 1.5	16.9 ± 0.2	5.1 ± 0.1	4.9 ± 0.1	18.0 ± 0.3	1.0 ± 0.0	0.005 ± 0.004	78.6 ± 0.3
24	31 ± 3	28 ± 3	24 ± 2	850 ± 77	1.9 ± 0.2	13.2 ± 0.2	6.3 ± 0.1	6.2 ± 0.1	17.8 ± 0.4	1.5 ± 0.0	0.016 ± 0.001	65.9 ± 0.4
25	38 ± 4	31 ± 3	25 ± 2	928 ± 84	2.8 ± 0.3	15.0 ± 0.1	7.5 ± 0.1	7.7 ± 0.1	22.0 ± 0.1	1.4 ± 0.0	0.019 ± 0.001	77.2 ± 1.5
26	11 ± 1	24 ± 2	19 ± 2	901 ± 81	15.0 ± 1.5	13.2 ± 0.3	6.2 ± 0.0	5.9 ± 0.0	18.4 ± 0.5	1.1 ± 0.0	0.017 ± 0.008	75.2 ± 1.8
27	27 ± 3	32 ± 3	27 ± 3	945 ± 85	2.6 ± 0.3	16.7 ± 0.3	6.3 ± 0.1	5.8 ± 0.1	18.4 ± 0.6	1.2 ± 0.0	0.005 ± 0.002	69.3 ± 1.8
28	23 ± 2	33 ± 3	28 ± 3	885 ± 80	2.0 ± 0.2	14.6 ± 0.2	7.1 ± 0.0	6.8 ± 0.1	18.1 ± 0.4	1.1 ± 0.0	0.009 ± 0.004	69.3 ± 0.3
29	7 ± 1	26 ± 2	22 ± 2	947 ± 85	1.8 ± 0.2	13.8 ± 0.2	5.6 ± 0.0	5.2 ± 0.1	15.3 ± 0.2	1.1 ± 0.0	0.000 ± 0.000	71.4 ± 0.6
30	40 ± 4	31 ± 3	25 ± 2	958 ± 86	0.2 ± 0.0	14.7 ± 0.1	6.3 ± 0.0	5.9 ± 0.0	17.1 ± 0.1	1.0 ± 0.0	0.008 ± 0.000	71.9 ± 0.1
31	34 ± 3	32 ± 3	26 ± 2	908 ± 82	2.7 ± 0.3	15.9 ± 0.2	6.8 ± 0.1	6.0 ± 0.1	18.9 ± 0.4	1.2 ± 0.0	0.007 ± 0.002	68.2 ± 0.1
32	23 ± 2	27 ± 3	26 ± 2	969 ± 87	6.7 ± 0.7	14.8 ± 0.3	6.7 ± 0.0	6.2 ± 0.1	19.2 ± 0.2	1.2 ± 0.0	0.013 ± 0.003	71.5 ± 1.1
33	33 ± 3	28 ± 3	23 ± 2	927 ± 83	6.1 ± 0.6	13.7 ± 0.1	6.4 ± 0.1	6.0 ± 0.1	18.2 ± 0.3	1.2 ± 0.0	0.016 ± 0.003	73.0 ± 0.3
34	12 ± 1	27 ± 3	23 ± 2	936 ± 84	3.4 ± 0.3	14.8 ± 0.3	6.9 ± 0.1	6.4 ± 0.1	19.0 ± 0.2	1.3 ± 0.0	0.004 ± 0.002	71.7 ± 0.4
35	34 ± 3	35 ± 3	27 ± 3	907 ± 82	2.1 ± 0.2	17.5 ± 0.1	9.6 ± 0.1	9.0 ± 0.1	23.3 ± 0.5	1.4 ± 0.0	0.015 ± 0.003	75.3 ± 0.5
36	15 ± 2	35 ± 3	30 ± 3	777 ± 70	5.3 ± 0.5	18.5 ± 0.3	10.4 ± 0.1	8.9 ± 0.1	25.3 ± 0.3	1.4 ± 0.0	0.023 ± 0.007	77.7 ± 1.8
37	33 ± 3	26 ± 2	22 ± 2	880 ± 97	13.1 ± 1.3	16.0 ± 0.3	6.6 ± 0.1	5.9 ± 0.1	21.5 ± 0.4	1.0 ± 0.0	0.025 ± 0.007	75.5 ± 0.2
38	34 ± 3	35 ± 3	29 ± 3	867 ± 78	4.8 ± 0.5	15.4 ± 0.2	6.9 ± 0.1	6.3 ± 0.1	19.3 ± 0.4	1.2 ± 0.0	0.009 ± 0.002	73.7 ± 1.7
39	35 ± 4	32 ± 3	28 ± 3	858 ± 77	10.7 ± 1.1	14.4 ± 0.2	7.4 ± 0.1	6.7 ± 0.1	20.8 ± 0.3	1.1 ± 0.0	0.017 ± 0.001	77.0 ± 0.5
40	18 ± 2	25 ± 2	21 ± 2	902 ± 81	13.0 ± 1.3	13.9 ± 0.2	7.0 ± 0.1	6.1 ± 0.1	19.3 ± 0.2	1.1 ± 0.0	0.015 ± 0.001	75.0 ± 0.2
41	22 ± 2	28 ± 3	24 ± 2	945 ± 85	2.7 ± 0.3	14.2 ± 0.2	6.2 ± 0.0	5.9 ± 0.1	17.7 ± 0.4	1.5 ± 0.0	0.003 ± 0.001	70.9 ± 1.7
42	19 ± 2	27 ± 2	23 ± 2	959 ± 86	0.4 ± 0.0	13.9 ± 0.2	5.9 ± 0.1	6.0 ± 0.1	16.7 ± 0.3	1.3 ± 0.0	0.003 ± 0.004	68.0 ± 0.4
43	23 ± 2	28 ± 3	24 ± 2	913 ± 82	1.9 ± 0.2	14.1 ± 0.3	5.8 ± 0.1	5.5 ± 0.1	17.0 ± 0.2	1.3 ± 0.0	0.001 ± 0.002	68.5 ± 0.5
44	16 ± 2	25 ± 2	21 ± 2	970 ± 87	7.3 ± 0.7	11.7 ± 0.1	5.2 ± 0.1	5.1 ± 0.1	15.4 ± 0.3	1.1 ± 0.0	0.009 ± 0.005	66.2 ± 0.9
45	34 ± 3	30 ± 3	26 ± 3	933 ± 84	1.7 ± 0.2	12.2 ± 0.2	5.3 ± 0.1	5.1 ± 0.1	17.1 ± 0.7	1.3 ± 0.0	0.005 ± 0.003	37.6 ± 0.4
46	31 ± 3	27 ± 2	24 ± 2	911 ± 82	9.8 ± 1.0	13.0 ± 0.3	12.6 ± 2.3	6.1 ± 0.1	14.1 ± 0.4	0.9 ± 0.0	0.010 ± 0.006	97.7 ± 2.9
47	31 ± 3	27 ± 2	24 ± 2	991 ± 89	0.6 ± 0.1	12.5 ± 0.4	7.7 ± 0.2	5.2 ± 0.1	12.8 ± 0.5	0.9 ± 0.0	0.006 ± 0.003	96.0 ± 1.1
48	19 ± 2	28 ± 3	22 ± 2	991 ± 89	0.4 ± 0.0	10.7 ± 0.3	6.1 ± 0.1	4.4 ± 0.1	10.1 ± 0.3	0.8 ± 0.0	0.002 ± 0.005	97.4 ± 1.6
Mean ± SD ^b^	26 ± 6	29 ± 3	25 ± 3	923 ± 47	5.6 ± 0.6	14.6 ± 1.9	5.4 ± 0.1	4.8 ± 0.0	18.1 ± 2.6	1.1 ± 0.2	0.009 ± 0.006	73.6 ± 1.0
MVC	40 ± 34	49 ± 3	37 ± 22	580 ± 202	-	61	26.9	22.6	74.2	11.2	0.097	26
CSG I						90	40	35	100	15	0.2	35
CSG II						300	50	100	250	25	0.6	300
WAV	35	45	32	412								

^a^ Activity concentration ± expanded uncertainty, ^b^ SD represents standard deviation; MVC: Mean values in China; CSG І: Chinese soil guidelines Grade І; CSG II: Chinese soil guidelines Grade II; WAV: world average values.

**Table 3:** The radiation hazard indices and lifetime cancer risk.

	Absorbed γ Dose Rate in Air (nGy/h)	Annual Effective Dose (μSv/y)	Radium Equivalent Activity (Bq/kg)	External Hazard Index	Internal Hazard Index	Lifetime Cancer Risk (1/Sv)
Mean ± SD	76.7 ± 3.1	83.1 ± 3.8	138.0 ± 6.8	0.37 ± 0.02	0.44 ± 0.03	3.2 × 10^−4^ ± 1.4 × 10^−5^
Median	67.9	83.3	138.78	0.37	0.44	3.2 × 10^−4^
Min-max	60.6–73.2	74.3–83.3	120.7–150.6	0.33–0.41	0.37–0.49	2.9 × 10^−4^–3.5 × 10^−4^

**Table 4:** Daily doses, hazard quotients, hazard indices, and cancer risks of heavy metals for children.

Heavy Metal		D_ing_	D_inh_	D_dermal_	HQ_ing_	HQ_inh_	HQ_dermal_	HI = ΣHQ	Cancer Risk
Cr	Mean	1.87 × 10^−4^	5.22 × 10^−^^9^	6.42 × 10^−^^6^	6.23 × 10^−^^2^	1.82 × 10^−^^4^	1.07 × 10^−^^1^	1.70 × 10^−^^1^	2.33 × 10^−^^7^
	Min	1.37 × 10^−4^	3.82 × 10^−^^9^	4.70 × 10^−^^6^	4.55 × 10^−^^2^	1.33 × 10^−^^4^	7.83 × 10^−^^2^	1.24 × 10^−^^1^	1.74 × 10^−^^7^
	Max	2.37 × 10^−4^	6.61 × 10^−^^9^	8.14 × 10^−^^6^	7.89 × 10^−^^2^	2.31 × 10^−^^4^	1.36 × 10^−^^1^	2.15 × 10^−^^1^	3.06 × 10^−^^7^
Ni	Mean	8.11 × 10^−^^5^	2.27 × 10^−^^9^	2.44 × 10^−^^5^	4.05 × 10^−^^3^	1.10 × 10^−^^7^	4.52 × 10^−^^3^	8.57 × 10^−^^3^	2.11 × 10^−^^7^
	Min	5.83 × 10^−^^5^	1.63 × 10^−^^9^	1.76 × 10^−^^5^	2.92 × 10^−^^3^	7.91 × 10^−^^8^	3.25 × 10^−^^3^	6.17 × 10^−^^3^	1.39 × 10^−^^7^
	Max	1.61 × 10^−4^	4.50 × 10^−^^9^	4.85 × 10^−^^5^	8.06 × 10^−^^3^	2.19 × 10^−^^7^	8.99 × 10^−^^3^	1.70 × 10^−^^2^	3.80 × 10^−^^7^
Cu	Mean	7.35 × 10^−^^5^	2.05 × 10^−^^9^	6.32 × 10^−^^6^	1.84 × 10^−^^3^	5.11 × 10^−^^8^	5.27 × 10^−^^4^	2.36 × 10^−^^3^	
	Min	5.56 × 10^−^^5^	1.55 × 10^−^^9^	4.78 × 10^−^^6^	1.39 × 10^−^^3^	3.86 × 10^−^^8^	3.98 × 10^−^^4^	1.79 × 10^−^^3^	
	Max	1.15 × 10^−4^	3.21 × 10^−^^9^	9.89 × 10^−^^6^	2.88 × 10^−^^3^	7.99 × 10^−^^8^	8.24 × 10^−^^4^	3.70 × 10^−^^3^	
Zn	Mean	2.31 × 10^−4^	6.45 × 10^−^^9^	3.97 × 10^−^^6^	7.70 × 10^−^^4^	2.15 × 10^−^^8^	6.62 × 10^−^^5^	8.36 × 10^−^^4^	
	Min	1.29 × 10^−4^	3.62 × 10^−^^9^	2.23 × 10^−^^6^	4.32 × 10^−^^4^	1.21 × 10^−^^8^	3.71 × 10^−^^5^	4.69 × 10^−^^4^	
	Max	3.23 × 10^−^^4^	9.04 × 10^−^^9^	5.56 × 10^−^^6^	1.08 × 10^−^^3^	3.01× 10^−^^8^	9.27 × 10^−^^5^	1.17 × 10^−^^3^	
As	Mean	1.46 × 10^−^^5^	4.08 × 10^−^^10^	3.77 × 10^−^^7^	4.87 × 10^−^^2^	1.36 × 10^−^^6^	3.06 × 10^−^^3^	5.17 × 10^−^^2^	2.33 × 10^−^^5^
	Min	1.02 × 10^−^^5^	2.85 × 10^−^^10^	2.64 × 10^−^^7^	3.41 × 10^−^^2^	9.49 × 10^−^^7^	2.14 × 10^−^^3^	3.62 × 10^−^^2^	1.63 × 10^−^^5^
	Max	1.98 × 10^−^^5^	5.53 × 10^−^^10^	5.11 × 10^−^^7^	6.60 × 10^−^^2^	1.84 × 10^−^^6^	4.15 × 10^−^^3^	7.01 × 10^−^^2^	3.16 × 10^−^^5^
Cd	Mean	1.15 × 10^−^^7^	3.21 × 10^−^^12^	1.38 × 10^−^^8^	1.15 × 10^−^^4^		1.38 × 10^−^^3^	1.50 × 10^−^^3^	7.44 × 10^−^^11^
	Min	0.00 × 10^0^	0.00 × 10^0^	0.00 × 10^0^	0.00 × 10^0^		0.00 × 10^0^	0.00 × 10^0^	0.00 × 10^0^
	Max	3.13 × 10^−^^7^	8.76 × 10^−^^12^	3.77 × 10^−^^8^	3.13 × 10^−^^4^		3.77 × 10^−^^3^	4.09 × 10^−^^3^	2.02 × 10^−^^10^
Pb	Mean	9.41 × 10^−4^	2.63 × 10^−^^8^	4.86 × 10^−^^6^	2.69 × 10^−^^1^	7.47 × 10^−^^6^	9.25 × 10^−^^3^	2.78 × 10^−^^1^	
	Min	4.80 × 10^−4^	1.34 × 10^−^^8^	2.48 × 10^−^^6^	1.37 × 10^−^^1^	3.81 × 10^−^^6^	4.72 × 10^−^^3^	1.42 × 10^−^^1^	
	Max	1.25 × 10^−^^3^	3.49 × 10^−^^8^	6.45 × 10^−^^6^	3.57 × 10^−^^1^	9.92 × 10^−^^6^	1.23 × 10^−^^2^	3.69 × 10^−^^1^	

**Table 5:** Daily doses, hazard quotients, hazard indices, and cancer risks of heavy metals for adults.

Heavy Metal		D_ing_	D_inh_	D_dermal_	HQ_ing_	HQ_inh_	HQ_dermal_	HI = ΣHQ	Cancer Risk
Cr	Mean	2.00 × 10^−^^5^	2.94 × 10^−9^	8.57 × 10^−7^	6.67 × 10^−3^	1.03 × 10^−4^	1.43 × 10^−^^2^	2.11 × 10^−^^2^	1.24 × 10^−7^
	Min	1.46 × 10^−^^5^	2.15 × 10^−9^	6.27 × 10^−7^	4.88 × 10^−3^	7.52 × 10^−5^	1.04 × 10^−^^2^	1.54 × 10^−^^2^	9.04 × 10^−^^8^
	Max	2.54 × 10^−^^5^	3.73 × 10^−9^	1.09 × 10^−6^	8.45 × 10^−3^	1.30 × 10^−4^	1.81 × 10^−^^2^	2.67 × 10^−^^2^	1.57 × 10^−7^
Ni	Mean	8.69 × 10^−^^6^	1.28 × 10^−9^	3.26 × 10^−6^	4.34 × 10^−4^	6.20 × 10^−^^8^	6.03 × 10^−4^	1.04 × 10^−3^	1.07 × 10^−7^
	Min	6.25 × 10^−^^6^	9.19 × 10^−10^	2.34 × 10^−6^	3.12 × 10^−4^	4.46 × 10^−^^8^	4.34 × 10^−4^	7.46 × 10^−4^	7.72 × 10^−^^8^
	Max	1.73 × 10^−^^5^	2.54 × 10^−9^	6.48 × 10^−6^	8.64 × 10^−4^	1.23 × 10^−7^	1.20 × 10^−3^	2.06 × 10^−3^	2.13 × 10^−7^
Cu	Mean	7.87 × 10^−^^6^	1.16 × 10^−9^	8.43 × 10^−7^	1.97 × 10^−4^	2.88 × 10^−^^8^	7.03 × 10^−5^	2.67 × 10^−4^	
	Min	5.96 × 10^−^^6^	8.76 × 10^−10^	6.38 × 10^−7^	1.49 × 10^−4^	2.18 × 10^−^^8^	5.32 × 10^−5^	2.02 × 10^−4^	
	Max	1.23 × 10^−^^5^	1.81 × 10^−9^	1.32 × 10^−6^	3.08 × 10^−4^	4.51 × 10^−^^8^	1.10 × 10^−4^	4.18 × 10^−4^	
Zn	Mean	2.47 × 10^−^^5^	3.64 × 10^−9^	5.30 × 10^−7^	8.25 × 10^−5^	1.21 × 10^−^^8^	8.83 × 10^−6^	9.13 × 10^−5^	
	Min	1.39 × 10^−^^5^	2.04 × 10^−9^	2.97 × 10^−7^	4.62 × 10^−5^	6.80 × 10^−9^	4.95 × 10^−6^	5.12 × 10^−5^	
	Max	3.47 × 10^−^^5^	5.10 × 10^−9^	7.42 × 10^−7^	1.16 × 10^−4^	1.70 × 10^−8^	1.24 × 10^−5^	1.28 × 10^−4^	
As	Mean	1.56 × 10^−^^6^	2.30 × 10^−10^	5.03 × 10^−^^8^	5.21 × 10^−3^	7.64 × 10^−7^	4.09 × 10^−4^	5.62 × 10^−3^	2.53 × 10^−6^
	Min	1.09 × 10^−^^6^	1.61 × 10^−10^	3.52 × 10^−^^8^	3.65 × 10^−3^	5.35 × 10^−7^	2.86 × 10^−4^	3.94 × 10^−3^	1.77 × 10^−6^
	Max	2.12 × 10^−^^6^	3.12 × 10^−10^	6.81 × 10^−^^8^	7.07 × 10^−3^	1.04 × 10^−6^	5.54 × 10^−4^	7.62 × 10^−3^	3.44 × 10^−6^
Cd	Mean	1.23 × 10^−^^8^	1.81 × 10^−12^	1.85 × 10^−9^	1.23 × 10^−5^		1.85 × 10^−4^	1.97 × 10^−^^4^	1.14 × 10^−^^11^
	Min	0.00 × 10^0^	0.00 × 10^0^	0.00 × 10^0^	0.00 × 10^0^		0.00 × 10^0^	0.00 × 10^0^	0.00 × 10^0^
	Max	3.36 × 10^−^^8^	4.94 × 10^−12^	5.04 × 10^−9^	3.36 × 10^−5^		5.04 × 10^−4^	5.37 × 10^−^^4^	3.11 × 10^−^^11^
Pb	Mean	1.01 × 10^−^^4^	1.48 × 10^−^^8^	6.48 × 10^−7^	2.88 × 10^−2^	4.21 × 10^−6^	1.23 × 10^−3^	3.01 × 10^−^^2^	
	Min	5.14 × 10^−^^5^	7.56 × 10^−9^	3.31 × 10^−7^	1.47 × 10^−2^	2.15 × 10^−6^	6.30 × 10^−4^	1.53 × 10^−^^2^	
	Max	1.34 × 10^−^^4^	1.97 × 10^−^^8^	8.60 × 10^−7^	3.82 × 10^−2^	5.59 × 10^−6^	1.64 × 10^−3^	3.99 × 10^−^^2^	

**Table 6:** The pearson correlation matrix for the natural radionuclides and the heavy metals.

	^238^U	^232^Th	^226^Ra	^40^K	Cr	Ni	Cu	Zn	As	Cd	Pb
^238^U	1	0.344 ^a^	0.333 ^a^	0.032	−0.002	0.192	0.224	0.261	0.173	0.146	0.045
^232^Th		1	0.933 ^b^	−0.367 ^a^	0.421 ^b^	0.298 ^a^	0.497 ^b^	0.512 ^b^	0.233	−0.035	0.0.35
^226^Ra			1	−0.303 ^a^	0.407 ^b^	0.297 ^a^	0.399 ^b^	0.446 ^b^	0.144	−0.111	0.040
^40^K				1	−0.009	−0.439 ^b^	−0.590 ^b^	−0.483 ^b^	−0.239	−0.619 ^b^	0.127
Cr					1	0.098	0.264	0.622 ^b^	0.13	0.082	0.219
Ni						1	0.756 ^b^	0.333 ^a^	0.179	0.414 ^b^	0.375 ^b^
Cu							1	0.737 ^b^	0.547 ^b^	0.544 ^b^	0.090
Zn								1	0.497 ^b^	0.538 ^b^	−0.032
As									1	0.168	−0.386 ^b^
Cd										1	0.092
Pb											1

^a^ Correlation is significant at the 0.05 level; ^b^ Correlation is significant at the 0.01 level.
